# A Case of Prostatic Metastasis from Intrahepatic Cholangiocarcinoma: An Extremely Rare Event

**DOI:** 10.7759/cureus.35100

**Published:** 2023-02-17

**Authors:** Sanathan Aiyadurai, Tulika Garg, Tass Sayeed, Zainab Shahbaz, Idowu O Adewole, Enoh Nguty Nkeng, Abia Joseph, Datiobong Udoeyop, Yusra Qamar, Aadil Khan

**Affiliations:** 1 Internal Medicine, Caribbean Medical University, Willemstad, CUW; 2 Medicine, Government Medical College & Hospital, Chandigarh, IND; 3 College of Medicine, Windsor University School of Medicine, Chicago, USA; 4 Research and Development, Windsor University School of Medicine, Chicago, USA; 5 College of Medicine, All Saints University School of Medicine Dominica, Roseau, DMA; 6 Public Health, DC Health, Washington, D.C., USA; 7 Surgery, John F. Kennedy University School of Medicine, Willemstad, CUW; 8 Surgery, Richmond Gabriel University, Chicago, USA; 9 Surgery, Lala Lajpat Rai Hospital, Kanpur, IND; 10 Internal Medicine, Lala Lajpat Rai Hospital, Kanpur, IND

**Keywords:** prostate cancer, lymphangiogenesis, chemoradiotherapy, metastasis, intrahepatic cholangiocarcinoma (icc)

## Abstract

The second most frequent primary carcinoma of the liver to emerge is intrahepatic cholangiocarcinoma (ICC), which is thought to be an incurable, rapidly proliferating tumor with a dismal prognosis. ICC is typically found at an advanced stage and is physiologically hostile. Regional lymph nodes and liver metastases are frequent tumor metastatic sites for ICC and serve as indicators of tumor recurrence. ICC metastasizing to the male urogenital tract has only seldom been documented. Typically, lymph vessels serve as the primary pathway for disseminating tumor cells. The high fatality rate associated with ICC and the rapid spread of the disease may be caused by this lymphatic route. The only curative therapeutic approach for treating these tumors is surgical removal. We report a case of prostatic metastasis from ICC.

## Introduction

ICCs (intrahepatic cholangiocarcinoma) are primary tumors typically arising from the epithelial lining of bile ducts within the parenchymal liver, adjacent to secondary biliary radicals, whereas extrahepatic ICCs arise from the liver's hilum ducts. The second most frequent liver cancer is ICC. Primary sclerosing cholangitis, hepatitis B and C, fatty liver disease, and diabetes are some risk factors for cholangiocarcinoma [1]. Most cholangiocarcinoma patients do not often have any underlying risk factors. While some individuals are asymptomatic, patients frequently report a history of weight loss and dull right upper quadrant pain. Most ICCs are adenocarcinomas histologically. According to reports, ICC metastases often occur in the liver, lung, bone, and brain [2]. It was found that cases of ICC metastasizing to the prostate were hardly recorded. Usually, lymph vessels act as the main channel for tumor cell spread. This lymphatic pathway may be the reason for the rapid disease progression and high mortality linked with ICC. Local expansion of the tumor cells from the primary tumor into the nearby lymphatics may be the primary driver of tumor-associated angiogenesis. Management is primarily surgery, and only a small percentage of patients (15%) have a resectable illness, with a median survival of fewer than three years [3]. Chemotherapy is another popular targeted therapy for incurable cancers [4].

## Case presentation

**Figure 1:**
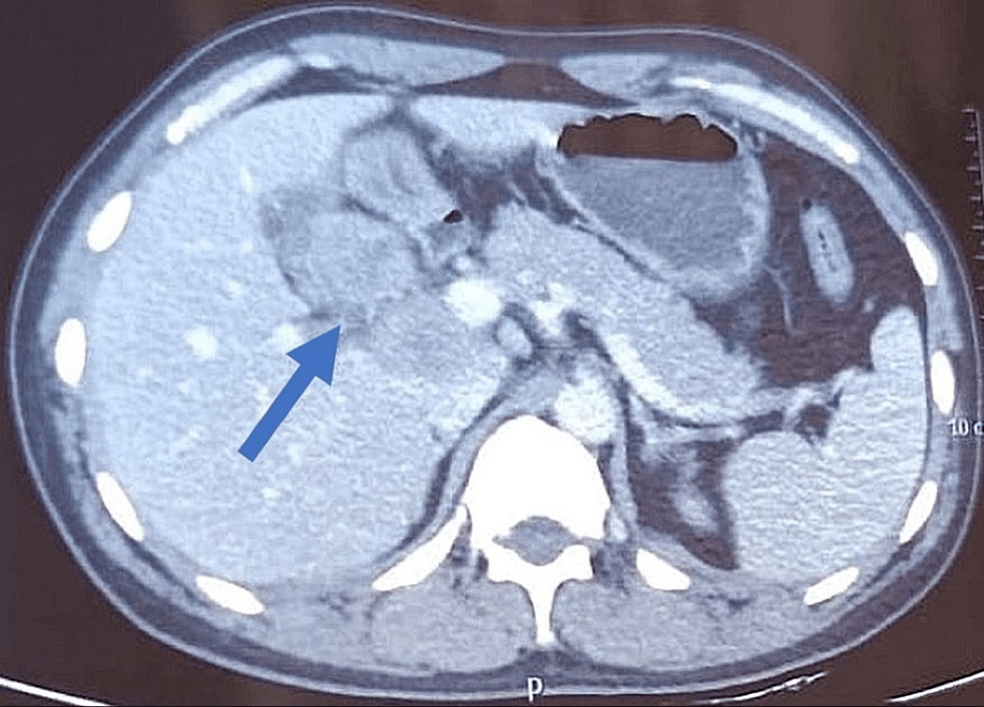
Heterogeneously enhanced irregular mass in the gallbladder body and fundus.

**Figure 2:**
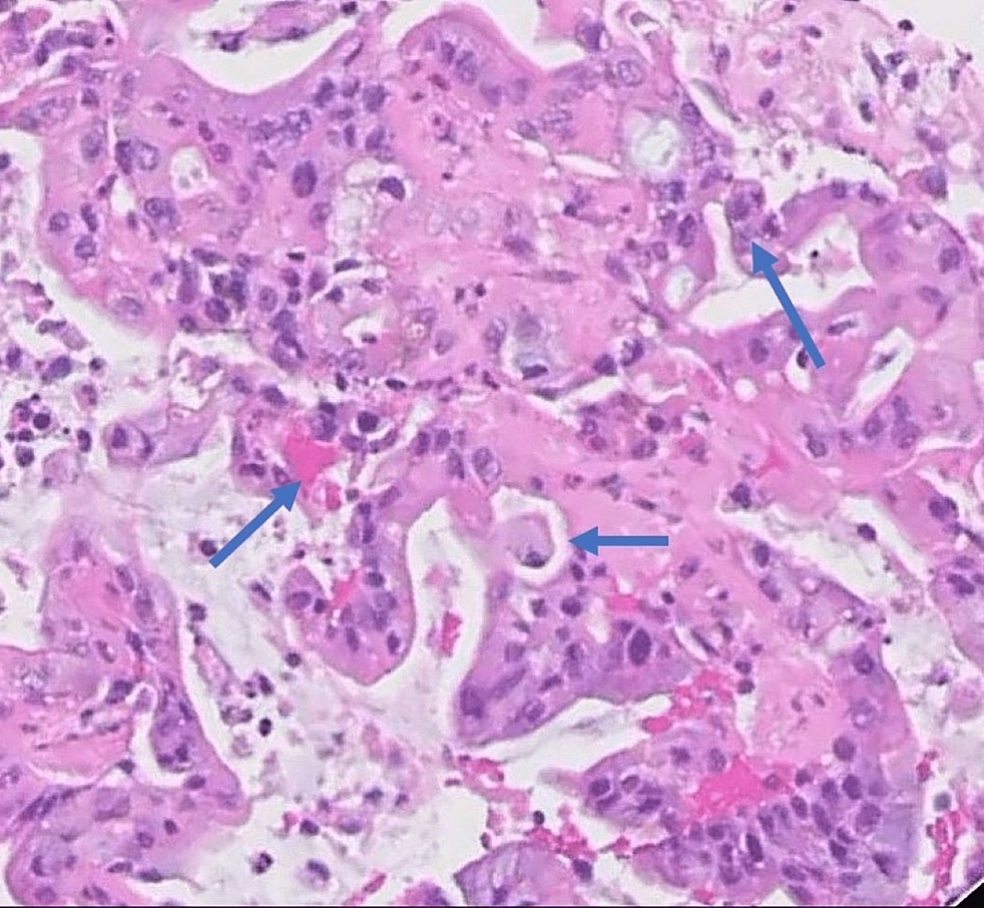
Histology specimen revealing enlarged pleomorphic nuclei, cytoplasm with focal mucin vacuoles, tumor cells with glandular formation, and their secretions in the lumina.

A 40-year-old male presented with chief complaints of anorexia, weight loss, and right-sided abdominal pain for the last two months. On general examination, the patient looked ill with mild icterus. He was afebrile and hemodynamically stable. Abdominal examination revealed mild tenderness in the upper right quadrant with a palpable mass. The rest of the physical examination was unremarkable. His biochemical and hematological parameters were within normal limits except for direct bilirubin which was increased to 2.3mg/dL (<0.3). On further investigation, computed tomography (CT) of the whole abdomen revealed an irregular mass in the gallbladder with large heterogeneously necrosed and enhanced lymph nodes in the periportal and portocaval regions with extensions (Figure 1). On further evaluation with endoscopic ultrasound-guided fine needle biopsy (EUS-FNB), gallbladder fundus mass revealed moderately cellular and tumor cells arranged in loose fragments and groups. Tumor cells showed enlarged pleomorphic nuclei and a moderate amount of cytoplasm with focal mucin vacuoles (Figure 2). Cellblock showed a few groups of similar tumor cells with glandular formation. The periportal lymph node showed fragments and groups of identical tumor cells with focal necrosis. He was diagnosed with cholangiocarcinoma with serum CA 19-9 marker of 801 U/ml.

**Figure 3:**
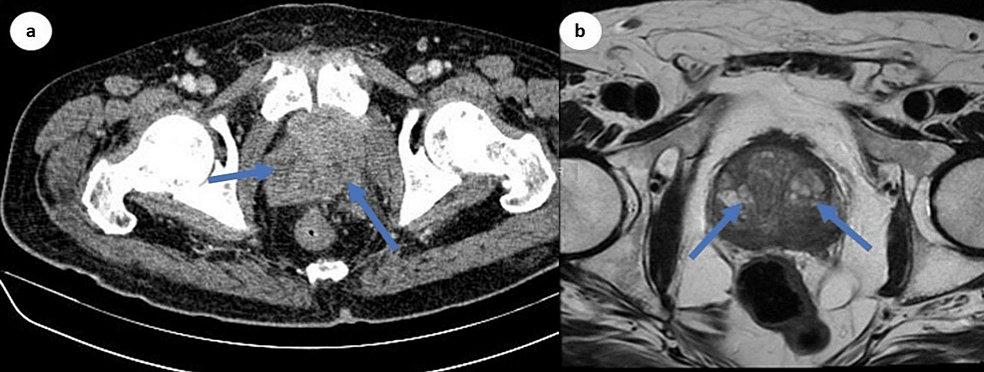
CT (a) and MRI (b) demonstrating multiple ill-defined lesions in the anterolateral regions of the prostate with irregular borders. CT: computed tomography; MRI: magnetic resonance imaging

He was planned to receive chemotherapy before liver transplantation; however, he refused chemotherapy and was discharged on symptomatic treatment. Three months later, he presented again with nocturia, urination hesitancy, and dull pain in the hips, which was not relieved by medication. His initial urine complete examination was positive for microscopic hematuria with no evidence of infection. Further evaluation revealed irregular prostate enlargement on digital rectal examination. He underwent pelvic CT followed by magnetic resonance imaging (MRI), which revealed ill-defined multiple lesions at the lower pelvis in the periphery of the urinary bladder around the urethra (Figure 3). During transurethral resection of the prostate, a small mass involving the right lobe of the prostate was resected and sent for histopathology, which confirmed perineural tumor and lymphangiosis carcinomatosis and metastases from primary cholangiocarcinoma.

After integrating the investigation findings and clinical features, a diagnosis of gallbladder carcinoma with metastasis to the prostrate was made; however, palliative care with chemotherapy was the management of choice and was advised. The patient was started on chemotherapy with gemcitabine and cisplatin every three weeks per cycle for up to four cycles and followed up with a CT scan of the abdomen every three months. The patient responded well to the treatment given. He has shown improvement and continues to be on follow-up.

## Discussion

ICC metastasizing to the urogenital areas was not widely reported, and only a few cases have been described. Tosev G et al. highlighted a case of ICC metastasizing to the prostate in a 79-year-old male [5]. Histopathology of the prostate revealed cholangiocarcinoma with negative prostate-specific antigen sensitivity. Another case of ICC with epidydimal metastases in an old male was also reported, who presented with obstructive jaundice and scrotal swelling [6].

ICC is viewed as an aggressive and fatal kind of cancer. Only the subgroup with minimal illness who had their tumors removed with clear margins showed long-term survival [7]. Worse outcomes and high mortality are associated with the large size of the tumor, multiple liver tumors, and tumor-positive perihepatic lymph node metastases [8]. Only individuals with minor illnesses who underwent resections with clear margins showed long-term survival. The prostate tissue's second pathology report confirmed prostatic metastases from ICC with the same cause [9]. If the prostate was the only site of the metastatic dissemination of tumor cells from a primary neoplasm, radiation therapy for the prostate in combination with adjuvant chemotherapy can be considered [10,11].

In cases where the resection margin status is positive or if progression takes place a few months after surgery, patients with metastatic ICC typically receive extra adjuvant therapy after surgery. With five-year survival rates between 5% and 15%, these tumors often have a poor prognosis [12]. Following primary surgical treatment and systemic adjuvant therapy [3], stereotactic radiofrequency ablation (SRFA) of the liver or chemoradiotherapy may be helpful to lower the risk of recurrence by focusing on any undetectable metastases in patients with high-risk features, such as those with node or margin positivity, particularly those with ICC [13]. The performance of transurethral resection of the prostate is advised in cases of intermittent hemorrhage due to enlarged varicose veins of the prostate, as well as to stop further local complications such as urinary retention, recurrent lower urinary tract infection, bladder tamponade, tumor anemia, and for diagnostic purposes. If the prostate was the only site of the metastatic dissemination of tumor cells from a primary neoplasm, prostate radiation therapy in conjunction with adjuvant chemotherapy may be an option [14].

It is unknown what role the tumor microenvironment plays in lymph nodes and prostate metastasis. In ICC, local expansion of the tumor cells from the primary tumor into the nearby lymphatics through penetration may be the primary driver of tumor-associated angiogenesis [15]. Common growth factors, including vascular endothelial growth factor (VEGF-C), which were overexpressed in both tumor types, can be secreted by both prostate cancer and ICC, and encourage lymph angiogenesis [16]. Hence, autocrine modulation of lymphangiogenic growth factors may enhance metastasis.

## Conclusions

ICC is a rapidly lethal form of cancer, and metastasis to the prostate is associated with high morbidity and mortality. Radiation therapy and adjuvant chemotherapy are potential treatments that could be considered if the prostate is the only site of metastatic dissemination from a primary tumor. This case highlights the rarity of prostrate metastases due to ICC and the option of caring for these metastases; nevertheless, further studies are required to explore and manage metastases related to ICC at an earlier stage.
